# Back pain in physically inactive students compared to physical education students with a high and average level of physical activity studying in Poland

**DOI:** 10.1186/s12891-017-1858-9

**Published:** 2017-11-28

**Authors:** Agnieszka Kędra, Aleksandra Kolwicz-Gańko, Przemysław Kędra, Anna Bochenek, Dariusz Czaprowski

**Affiliations:** 1 Jozef Pilsudski University of Physical Education in Warsaw, Faculty of Physical Education and Sport, ul. Akademicka 2, 21-500 Biala Podlaska, Poland; 2 Jozef Rusiecki University College in Olsztyn, Faculty of Physiotherapy, ul. Bydgoska 33, 10-243 Olsztyn, Poland; 3 Jozef Pilsudski University of Physical Education in Warsaw, Faculty of Tourism and Sport, ul. Akademicka 2, 21-500 Biala Podlaska, Poland

**Keywords:** Pain, Spine, Students, Trained individuals, Physical activity

## Abstract

**Background:**

The aim of the study was (1) to characterise back pain in physically inactive students as well as in trained (with a high level of physical activity) and untrained (with an average level of physical activity) physical education (PE) students and (2) to find out whether there exist differences regarding the declared incidence of back pain (within the last 12 months) between physically inactive students and PE students as well as between trained (with a high level of physical activity) and untrained (with an average level of physical activity) PE students.

**Methods:**

The study included 1321 1st-, 2nd- and 3rd-year students (full-time bachelor degree course) of Physical Education, Physiotherapy, Pedagogy as well as Tourism and Recreation from 4 universities in Poland. A questionnaire prepared by the authors was applied as a research tool. The 10-point Visual Analogue Scale (VAS) was used to assess pain intensity. Prior to the study, the reliability of the questionnaire was assessed by conducting it on the group of 20 participants twice with a shorter interval. No significant differences between the results obtained in the two surveys were revealed (*p* < 0.05).

**Results:**

In the group of 1311 study participants, 927 (70.7%) respondents declared having experienced back pain within the last 12 months. Physically inactive students declared back pain frequency similar to the frequency declared by their counterparts studying physical education (*p* > 0.05). Back pain was more common in the group of trained students than among untrained individuals (*p* < 0.05). Back pain was mainly located in the lumbar spine.

**Conclusions:**

A frequent occurrence of back pain (70.7%) was noted in the examined groups of students. The percentage of students declaring back pain increased in the course of studies (p < 0.05) and, according to the students’ declarations, it was located mainly in the lumbar spine. No significant differences regarding the incidence of back pain were found between physically inactive students and physical education students (*p* > 0.05). The trained students declared back pain more often than their untrained counterparts (*p* < 0.05).

**Electronic supplementary material:**

The online version of this article (10.1186/s12891-017-1858-9) contains supplementary material, which is available to authorized users.

## Background

Back pain is a common health issue that constitutes the main reason for sick leaves and disability retirement of working individuals, which, in turn, generates considerable costs in the healthcare system [1–3]. The results of the studies from the last 25 years indicate that back pain is declared not only by adults and elderly individuals but also by adolescents and even school children [4–6]. Risk factors predisposing young people to back pain mainly include low body height [7], female gender [4, 5, 8–10], mental disorders [11], and depression and stress [4]. Modifiable risk factors include smoking tobacco [4], time spent in front of the TV [5], and high or low levels of physical activity [12, 13]. Studies revealed that physical activity is significant for preventing and treating back pain [14–18]. However, both passivity and excessive physical activity may increase the risk of back pain [12]. Numerous studies carried out on groups of young individuals doing sports revealed that back pain is a common phenomenon [19–22]. To date, back pain in athletes has been analysed in comparison to the group of physically inactive individuals; however, correlations between back pain and physical activity at different levels have not been verified. Therefore, it is significant to complete the current body of knowledge with such an analysis.

Physical education students constitute a specific group of individuals with a moderate and high level of physical activity. They take up physical activity within their obligatory physical education classes which are included in the study curriculum. Moreover, a considerable group of students of physical education are athletes who have been training various individual and team sports for many years.

The aim of the study was (1) to characterise back pain in physically inactive students as well as trained and untrained PE students and (2) to find out whether there exist differences regarding the declared incidence of back pain (within the last 12 months) between physically inactive students and PE students as well as between trained and untrained PE students.

## Methods

### Study participants

The study included 1321 1st-, 2nd- and 3rd-year students (full-time bachelor degree course) of Physical Education, Physiotherapy, Pedagogy as well as Tourism and Recreation from 4 universities in Poland. The sample was randomly selected with the use of multistage cluster design [23]. The first stage included a random selection of universities from particular regions of eastern Poland that educate students in various fields, one of them necessarily being Physical Education. The second stage involved the selection of two fields of study at each university. At the final (third) stage, student groups (B.A. students) were selected from each field of study. The research included all the students who attended classes on the day of conducting the research (it was not repeated in the case of absent students). Students who gave their consent to participate in the research were qualified for it.

The final analysis included 1311 questionnaires constituting 99.2% of the study population. Taking into account the obtained questionnaires, the study participants were divided into two groups, i.e. (1) physically inactive students and (2) PE students. Moreover, the latter group was divided into two subgroups, i.e. (a) untrained PE students – an average level of physical activity and (b) trained PE students – a high level of physical activity. The inclusion criteria in the group of physically inactive students were as follows: taking one course only; being a student of the field of study other than physical education (or any other course connected with physical activity); studying at the 1st, 2nd or 3rd year of full-time bachelor degree course; attending no additional physical education classes apart from PE included in the curriculum; undergoing no sports training at the time of the research or in the past; taking up free time physical activity no more than once per week and no longer than 60 min. The inclusion criteria in the group of untrained PE students – an average level of physical activity: taking one course only; studying at the 1st, 2nd or 3rd year of a PE course (Bachelor’s degree); physical education classes constituting a minimum of 40% of the course curriculum (798 h in the period of 3 years); undergoing no training at the time of the research or in the past. In a 3-year curriculum PE students attend the following obligatory sports classes: gymnastics, rhythmic gymnastics, dancing, swimming, athletics, handball, football, volleyball, basketball, motor games and plays, wrestling, fitness, weight training, winter camp (with such classes as alpine skiing and cross-country skiing), summer camp (with such classes as windsurfing, canoeing, field games and plays, survival, cycling), optional sports specialisation. Within three years of studying (six semesters) each student attends 798 h of sports classes. The inclusion criteria in the group of trained PE students – a high level of physical activity: taking one course only; studying at the 1st, 2nd or 3rd year of a PE course (Bachelor’s degree); physical education classes constituting a minimum of 40% of the course curriculum (798 h in the period of 3 years); training a minimum of 60 min per day – 5 times per week, training experience – a minimum of 3 years, break from training within the last year no longer than 1 week. The group of trained students included individuals who trained one of the following team sports: handball, football, volleyball or basketball.

Individuals taking more than one course were excluded from the study, since four PE students additionally studied a course which had no physical activity in the curriculum (pedagogics, geography) and including these individuals in the analysis might mean that the same student could be counted in two different groups. Moreover, one student experiencing pain only during pregnancy and two students feeling pain only during menstruation were excluded from the study, since there is evidence that these ailments experienced by women in these periods may result, inter alia, from endocrine disruptions and it is difficult to qualify them unequivocally as back pain [24].

### Diagnostic tool

A questionnaire prepared by the authors was applied as a research tool. All the students filled in the questionnaire during classes at the university (practical classes or lectures) with one of the study authors present. The first page of the questionnaire included an explanation of the study aim and instructions. The questionnaire consisted of multiple choice questions allowing respondents to select only one answer (14 questions) or multiple answers (2 questions). Moreover, 9 multiple choice questions included a comment section. Personal information section consisted of questions about university course or courses, age, year of studying, gender, body mass and height, training (sport, number of training days per week, number of training hours per day).

The main section of the questionnaire included questions regarding:

- experiencing back pain within the last year (12 months). Individuals who responded negatively to this question, were asked not to answer the remaining questions;
- pain frequency, location and intensity;
- types of situations in which back pain occurred or increased (Additional file 1).

In order to assess the intensity of pain, Visual Analogue Scale (VAS) was applied. The subject’s task was to mark their maximal pain level from the previous month on a 10-cm line. Then, the centimetres marked by the respondents were measured and converted into point scale and classified according to the following key: 0 – no pain, 1-3 – mild pain, 4-6 – moderate pain, 7-10 – severe pain [25].

Prior to the study, the reliability of the questionnaire was assessed by conducting the survey twice in the group of 20 participants (5 persons from each of the four courses) with a 1-month interval. Kappa coefficient in all the variables was equal to or higher than 0.93. No significant differences between the results obtained in both tests (*p* < 0.05) were found.

The questionnaire was anonymous and voluntary. The study was accepted by the Ethical Commission of Scientific Research of Jozef Pilsudski University of Physical Education in Warsaw, Poland.

### Statistical methods

The analysis was made with the use of descriptive statistics. A non-parametric Chi-square test was applied. The odds ratio (OR) and corresponding 95% confidence interval was calculated. Calculations were made with the use of SPSS 9.0 software and Excel spread sheet. The level of significance was set at alfa <0.05.

## Results

### Declared back pain occurrence

In the group of 1311 study participants, 927 (70.7%) respondents declared having experienced back pain within the last 12 months. Taking into account the nature of the course, it may be concluded that physically inactive students declared back pain at a frequency similar to that declared by their counterparts studying PE (70.4 and 71.2%, respectively), *p* > 0.05 (OR CI 1.0 95% 0.82-1.32). Back pain is more common among the trained students than their untrained peers (75.3 and 67.8%, respectively) and this is a statistically significant difference at the level of *p* < 0.05. It is also confirmed by the value of the odds ratio, i.e. 1.5 (95% CI 0.99-2.1) (Table 1). In all the examined groups of students, the declared occurrence of back pain increased in the course of studies (p < 0.05) (Table 1).

**Table 1:** Incidence of BP with regard to the year of studies depending on the active or inactive character of studies (*n* = 1311)

	Students		Students					PE students				
	T (PIS/PES)		PIS			TPES (UT/T)		UT			T	
	n	%	n	%	*p* value	n	%	n	%	*p* value	n	%
BP incidence												
TOTAL	(n = 1311)		(*n* = 739)			(*n* = 572)		(*n* = 317)			(*n* = 255)	
No	384	29.3	219	29.6	(NS)	165	28.8	102	32.2	<0.05	63	24.7
Yes	927	70.7	520	70.4		407	71.2	215	67.8		192	75.3
BP incidence With regard to the year of studies												
1st-year students	(*n* = 462)		(*n* = 278)			(*n* = 184)		(*n* = 105)			(*n* = 79)	
No	186	40.3	112	40.3	(NS)	74	40.2	46	43.8	(NS)	28	35.4
Yes	276	59.7	166	59.7		110	59.8	59	56.2		51	64.6
2nd-year students	(*n* = 510)		(*n* = 294)			(*n* = 216)		(*n* = 115)			(*n* = 101)	
No	163	32.0	84	28.6	(NS)	79	36.6	49	42.6	<0.05	30	29.7
Yes	347	68.0	210	71.4		137	63.4	66	57.4		71	70.3
3rd-year students	(*n* = 339)		(*n* = 167)			(*n* = 172)		(*n* = 97)			(*n* = 75)	
No	35	10.3	23	13.8	<0.05	12	7.0	7	7.2	(NS)	5	6.7
Yes	304	89.7	144	86.2		160	93.0	90	92.8		70	93.3
*p* value	<0.05		<0.05			<0.05		<0.05			<0.05	

*BP* back pain, *NS* statistically insignificant, *PIS* physically inactive students, *PES* physical education students, *UT* untrained PE students, *T* trained PE students, *T* total, *TPES* all PE students

### Declared back pain incidence

Having analysed the declared incidence of back pain, it may be concluded that the biggest group is constituted by respondents who experienced pain rarely, i.e. 1–2 times per year (53.4%). While analysing the frequency of back pain with regard to the type of studies, it was noted that physically inactive students and PE students declared a similar frequency of back pain, i.e. very rare pain (1-2 times per year) – 54.8 and 51.6%, respectively, and pain occurring a few times per year (3-6 times/year) – 34.6 and 31.0%, respectively. Frequent and constant pain (more than 1-2 times per month) was declared more often by PE students than by physically inactive students (10.6 and 17.4%, respectively) (*p* < 0.05). Pain occurring 1-2 times per year was more common among untrained students than among their trained counterparts, while frequent or constant pain was declared more often by trained students than by untrained ones. However, the difference was not statistically significant (*p* > 0.05) (Table 2).

**Table 2:** Incidence, location, intensity and circumstances of BP in the group of students depending on the active or inactive character of studies (*n* = 927)

	Students T (PIS/PES)		Students PIS					PE students				
						TPES (UT/T)		UT			T	
	(n = 927)		(*n* = 520)			(*n* = 407)		(*n* = 215)			(*n* = 192)	
	n	%	n	%	*p* value	n	%	N	%	*p* value	n	%
BP incidence												
Rare BP (1-2/year)	495	53.4	285	54.8	<0.05	210	51.6	120	55.8	(NS)	90	46.9
BP several times per year (3-6/year)	306	33.0	180	34.6		126	31.0	59	27.4		67	34.9
Frequent or constant BP (more than 1-2 months)	126	13.6	55	10.6		71	17.4	36	16.7		35	18.2
BP location (segment) ^a^												
Cervical	176	19.0	128	24.6	<0.05	48	11.8	30	14.4	(NS)	18	9.4
Thoracic	167	18.0	100	19.2	(NS)	67	16.5	39	18.7	(NS)	28	14.6
Lumbar	810	87.4	473	91.0	<0.05	337	82.8	176	84.2	(NS)	161	83.9
BP intensity												
Mild	337	36.4	198	38.1	<0.05	139	34.2	86	41.1	<0.05	53	27.6
Moderate	392	42.3	230	44.2		162	39.8	82	39.2		80	41.7
Severe	198	21.4	92	17.7		106	26.0	47	19.6		59	30.7
Circumstances in which BP occurs or increases ^a^												
Sitting	457	49.3	308	59.2	<0.05	149	36.6	87	41.6	(NS)	62	32.3
Standing	335	36.1	203	39.0	<0.05	132	32.4	74	35.4	(NS)	58	30.2
Lying	150	16.2	94	18.1	(NS)	56	13.8	31	14.8	(NS)	25	13.0
Lifting heavy objects	246	26.5	143	27.5	(NS)	103	25.3	57	27.3	(NS)	46	24.0
Performing everyday activities (cleaning, cooking, getting dressed)	222	23.9	143	27.5	<0.05	79	19.4	42	20.1	(NS)	37	19.3
Physical effort	229	24.7	112	21.5	<0.05	117	28.7	56	26.8	(NS)	61	31.8
Don’t remember	81	8.7	38	7.3	–	43	10.6	25	12.0	–	18	9.4
Other	51	5.5	34	6.5	–	17	4.2	8	3.8	–	9	4.7

*BP* back pain, *NS* statistically insignificant, *PIS* physically inactive students, *PES* physical education students, *UT* untrained PE students, *T* trained PE students, *T* total, *TPES* all PE students

^a^It does not add up to 100% as the respondents were allowed to mark more than one answer /*

### Back pain location

The question concerning pain location was a multiple choice question allowing more than one answer. Back pain was mainly located in the lumbar spine. This location was declared by 87.4% of the respondents. The analysis of pain location with regard to the type of studies revealed that physically inactive students declared pain in the cervical and lumbar spine more often than PE students (*p* < 0.05) (Table 2).

### Back pain intensity

The analysis of back pain intensity allowed us to conclude that moderate pain was the most common in the examined group as it was declared by 42.3% of the respondents. As for pain intensity with regard to the type of studies, physically inactive students declared mild and moderate pain (38.1 and 44.2%, respectively) more often than PE students (34.2 and 39.8%, respectively) (*p* < 0.05). Severe pain was more often declared by PE students than by physically inactive students (26.0 and 17.7%, respectively). The analysis of back pain intensity in the group of PE students showed that untrained students declared mild pain more often than trained students (41.1 and 27.6%, respectively), while severe pain occurred more often in trained students than among untrained ones (30.7 and 19.6%, respectively) (p < 0.05). Detailed data are presented in Table 2.

### Circumstances in which back pain occurred or increased

The question concerning circumstances in which back pain occurred or increased was a multiple choice question allowing more than one answer. The most common circumstances in which back pain occurred or increased included sitting (49.3%) and standing (36.1%). While analysing the circumstances with regard to the type of studies, it was noted that in such circumstances as sitting (59.2%), standing (39.0%) and doing household chores (29.5%) back pain occurred and increased in physically inactive students more often than in PE students (*p* < 0.05). Physical effort caused or increased pain more often in PE students than in physically inactive students (28.7 and 21.5%, respectively) (p < 0.05). The study revealed no differences (*p* > 0.05) regarding circumstances in which back pain occurred or increased between the groups of untrained and trained students (Table 2).

## Discussion

In the available literature of the subject we have found studies which analysed the occurrence of back pain among students; however, these are studies analysing only low back pain. We did not limit our study to low back pain but we took into account all segments of the spine. Moreover, in the analysis attention was paid to the specificity of physical education studies which allows for selecting individuals with various levels of physical activity (a high level of physical activity – trained students, average level of physical activity – untrained students).

Our research revealed that back pain (which occurred within the last 12 months) affected a considerable group (70.7%) of students from Poland. Physically inactive students declared an incidence of back pain at a level similar to PE students (70.4 and 71.2%, respectively), *p* > 0.05. The similarity of the frequency of occurrence of back pain in physically inactive students and PE students may be related observations made by Heneweer et al. [12]. Their research conducted on 3664 randomly selected individuals over 25 years of age proved that the correlation between back pain and physical activity may be U-shaped. Both a sedentary lifestyle and high physical activity increased the risk of back pain [12].The authors drew attention to the fact that it is not the quantity of physical activity that is significant but its quality. A boundary between moderate physical activity and excessive activity also depends on physical fitness. It has to be highlighted that it is significant whether an individual is forced to take up physical activity or if it is voluntary [12]. On the other hand, the lack of differences between the examined groups regarding the declared frequency of occurrence of back pain definitely suggests that a deeper analysis of factors connected with the lifestyle of the respondents is necessary. Our research revealed that there are differences between the compared groups regarding circumstances in which back pain occurs.

In our research, declared pain was mainly located in the lumbar spine (87.4%). The analysis of pain intensity showed that moderate pain occurred commonly. It can be noted that untrained students declared mild back pain more often than trained students (41.1 and 27.6%, respectively), while severe pain occurred more often in trained students than in untrained ones (30.7 and 19.6%) *p* < 0.05. It may be assumed that a higher percentage of trained students declaring severe and strong pain might mean that the problem results from an improper training process (e.g. too big training loads, improper selection of exercises). Verification of such assumptions requires detailed analysis of training aspects; however, it is not the aim of this work.

The research by Lewandowski et al. (2011) on 461 Polish PE and physiotherapy students revealed an incidence of back pain (77 and 69%, respectively) similar to our results. However, they analysed only low back pain and limited their analysis to 1st-year students only [26]. The research on 514 physically inactive Turkish students aged 17-29 years from the Faculty of Medicine, Engineering, Science-Literature, and Education revealed that back pain was experienced by 44.6% of the students from the Faculty of Medicine, 16.9% from the Faculty of Engineering, 20.2% from the Faculty of Education and 18.3% from the Faculty of Science-Literature [27]. This Turkish study indicated a significantly lower incidence of back pain than in the case of Polish physically inactive students. It may result from the fact that the quoted study focused on the analysis of back pain risk factors and both sample selection criteria and inclusion criteria differed from our study. Similarly to the previous study, in this research only non-specific low back pain was analysed.

Our own research revealed that trained students experienced back pain more often than untrained students (75.3 and 67.8%, respectively) (*p* < 0.05).

The incidence of back pain has been examined in several studies in the last few years [28–30]. The authors analysed thoraco-lumbar back pain in various sports (e.g. gymnastics – 67%, water ski jumping – 45%, football – 53%, weightlifting – 71%, wrestling – 77%, hockey – 89%, diving – 89% and tennis – 50%) in which considerable spine loads may occur, and found a significant percentage of individuals declaring back pain [28–31]. It was also revealed that back pain occurred more often in sports and competitions which require substantial (especially axial) spinal loads as well as among untrained individuals [30–35].

The research on back pain in physically inactive students and PE students from Poland may contribute to the findings of research concerning this issue in other countries. The obtained results may serve as a stimulus for further research aimed at finding back pain risk factors.

### Study limitations and strengths

A group of trained individuals included students who trained various team sports. Each of the sports has a specific type of training, which may have affected the occurrence and the location of back pain in athletes training particular sports. Another study limitation is that the study participants were not divided into gender groups for the analysis. It resulted from the fact that such a division would have brought about overrepresentation of women from the physically inactive group. It is related to the demographic structure of the group of PE students where females are in a minority. The fact that the study did not analyse free-time activities, number of hours spent in a sitting position, ways of sitting or other daily activities of the study participants which may have exerted either positive or negative influence on pain incidence is another study limitation. Another drawback resulting from the methodology of a cross-sectional study is the lack of possibility to determine the cause and effect correlation between factors affecting pain and its effects. Only the fact that certain correlations exist was determined. Due to the fact that respondents were asked about detailed characteristics of their back pain within the last 12 months, the final analysis of the results should be carried out with certain caution. However, the questionnaire applied in our study is reliable and according to authors, it may be used in clinical practice.

A big sample group, random selection of students and study group uniformity are the study strengths. A high response rate was achieved (99.2%). Information concerning physical activity and sport was gathered independently so as not to suggest further correlation. To the authors’ knowledge, it is the first study which analyses the characteristics of back pain in students with regard to the character of studies (active or sedentary) and additionally takes into account the division into trained and untrained PE students.

### Perspectives

In the examined group of trained students, a considerable percentage of participants declaring back pain was found. Further research should aim to analyse this group more broadly in terms of back pain characteristics and to find back pain risk factors. In the future, pain with regard to particular sports, training experience and the number of training days and hours should be analysed.

## Conclusions

1. In the examined groups of physically inactive and PE students a very frequent occurrence of back pain (70.7%) was noted. The percentage of students declaring back pain increased in the course of studies (*p* < 0.05) and according to the students’ declarations, it was located mainly in the lumbar segment of the spine (87.4%).
2. Physically inactive students most often declared mild and moderate pain. PE students declared severe and strong pain more often than physically inactive students (p < 0.05).
3. No significant differences regarding the incidence of back pain were found between physically inactive students and PE students (*p* > 0.05). The trained students declared back pain more often than their untrained counterparts (p < 0.05).

## Additional file

Additional file 1: questionnaire in English – questions concerning the feeling and characteristics of back pain in the English language. (DOC 70 kb)

## Abbreviations

PE: Physical education
VAS: Visual analogue scale
