# Supplementary material for: Back pain in physically inactive students compared to physical education students with a high and average level of physical activity studying in Poland
Source: BMC Musculoskelet Disord. 2017 Nov 28;18:501. doi: 10.1186/s12891-017-1858-9 (PMC5706389; doi:10.1186/s12891-017-1858-9)
Supplement: Additional file 1: — questionnaire in English – questions concerning the feeling and characteristics of back pain in the English language. (DOC 70 kb) [file 12891_2017_1858_MOESM1_ESM.doc]

**QUESTIONNAIRE**

***Dear Participants,***

*We would like to ask you to answer the following questions. The questionnaire is anonymous.*

*The aim of the study is to collect information regarding the frequency of occurrence and risk factors of back pain. The results will be used only for scientific purposes.*

*In the questions marked with * you may choose more than one answer, in the remaining questions only one answer can be selected.*

**Thank you**

**Year of studies**........................**Field(-s) of study/University**

.................................................................................................................................................................................................................................................................................................................................................................................................................

**Sex**: Female/Male **Date of birth** day.....................month...........................year......................

**Body height** (cm)........................................**Body mass** (kg).......................................................

**1. What is the character of your studies?**

a) mainly sedentary

b) sedentary, 1 or 2 times per week I’ve got practical classes during which I don’t sit

c) active character, a minimum of 3-4 times per week I’ve got sports classes

**2. Are any sports classes obligatory in your curriculum?**

a) no

b) yes (what type and how many hours per week?)

......................................................................................................................................................................................................................................................................................................................................................................................................................................................................................................................................................................................................

**3. Are you a professional athlete in any sport?**

a) no (if you chose “no”, please go to question no 9)

b) yes

**4. What sport do you do?**.....................................................................................................................................

**5. How many hours per day do you train?**..................................................................................................................................

**6. How many times per week do you train?**................................................................................................................................

**7. For how many years have you been training?.**......................................................................................................................

**8. Did you have a break from training longer than 1 week within the last year?**

a) ye, I did

b) no, I didn’t

**9. How much time per week do you devote to physical activity in your free time?**

a) more than 4 hours

b) approximately 2-3 hours

c) less than one hour

d) none

**10. How often do you walk?**

a) I don’t walk at all

b) rarely (2 or 3 times per month), I go by car/public transport more often

c) I often walk (2-3 times per week)

d) a lot, I walk almost every day

**11. Have you experienced back pain for the last year (12 months)?**

1. no, never
2. yes, rarely (1-2 times/year)
3. yes, a few times per year (3-6 times/year)
4. yes, very often or constantly (more than 1-2 times/month)
5. Yes, but only during menstruation

Individuals who chose answer ‘a) no, never’ in the above question (no 11), don’t answer the following questions. Thank you!

**12.** **Back pain is located in:***

a) the cervical spine

b) the thoracic spine

c) the lumbar spine

**13. In what circumstances does the back pain usually occur? (fill in the segment of the spine in which the pain occurs) ***

a) while sitting for a long time (in the .....................spine)

b) while standing (in the .....................spine)

c) while lying (in the .....................spine)

d) while lifting heavy objects (in the .....................spine)

e) while doing household chores (cleaning, cooking, getting dressed, etc.) (in the .....................spine)

f) during physical effort (in the .....................spine)

g) I don’t remember

h) other (specify) ...................................................................................................................................................................

**14. At the 10-centimetre line, please mark the intensity of the pain (the most severe one) that you experienced within the last month (0 – means no pain, 10 – means pain which is hard to bear)? (in the .....................spine)**

**
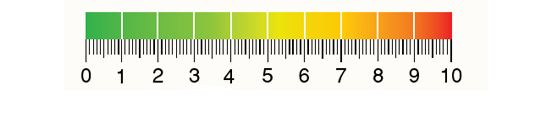
**
